# Milky serum during pregnancy

**DOI:** 10.11604/pamj.2025.50.10.46009

**Published:** 2025-01-06

**Authors:** Thiti Snabboon, Supanit Puttipokin

**Affiliations:** 1 Department of Medicine, Faculty of Medicine, Chulalongkorn University, Bangkok, Thailand,; 2 Excellence Center in Diabetes, Hormone and Metabolism, King Chulalongkorn Memorial Hospital, Thai Red Cross Society, Bangkok, Thailand,; 3 Public Health Center, Bangkok Metropolitan Administration, Bangkok, Thailand

**Keywords:** Lipemic, hypothyroidism, pregnancy, milky serum

## Image in medicine

A 37-year-old primigravida woman at 40 weeks gestation presented with milky serum during a preoperative evaluation for normal labor. The patient had no history of recurrent abdominal pain, and her previous annual check-ups had shown normal serum lipid and plasma glucose levels. Laboratory tests revealed severe hypertriglyceridemia (5,135 mg/dL), while serum amylase and lipase levels were normal. Additional physical findings showed a puffy face, bradycardia, and goiter, raising suspicion of hypothyroidism due to Hashimoto´s thyroiditis, which was confirmed through hormonal and thyroid antibody analysis. After a brief fasting period and initiation of L-thyroxine therapy, her triglyceride levels significantly decreased to 867 mg/dL. The patient subsequently delivered a healthy baby, and her triglyceride levels normalized postpartum without the need for lipid-lowering medication. Milky or lipemic serum, characterized by a white and creamy appearance, indicates a severe form of hypertriglyceridemia (exceeding 1,000 mg/dL) and should be distinguished from cloudiness caused by bacterial contamination. This condition can lead to serious complications, including acute pancreatitis or hyperviscosity syndrome. Pregnancy and hypothyroidism are well-known exacerbating factors in individuals with a genetic predisposition to hypertriglyceridemia. Other potential causes include poorly controlled diabetes, alcoholic consumption, nephrotic syndrome, and certain medications. Management involves correcting underlying medical conditions and a comprehensive approach, including supervised fasting or fat-restricted diets, medium-chain triglycerides supplementation, insulin therapy, and triglyceride-lowering medications such as fibrate and omega-3 fatty acids. In severe cases, plasmapheresis should be considered.

**Figure 1:**
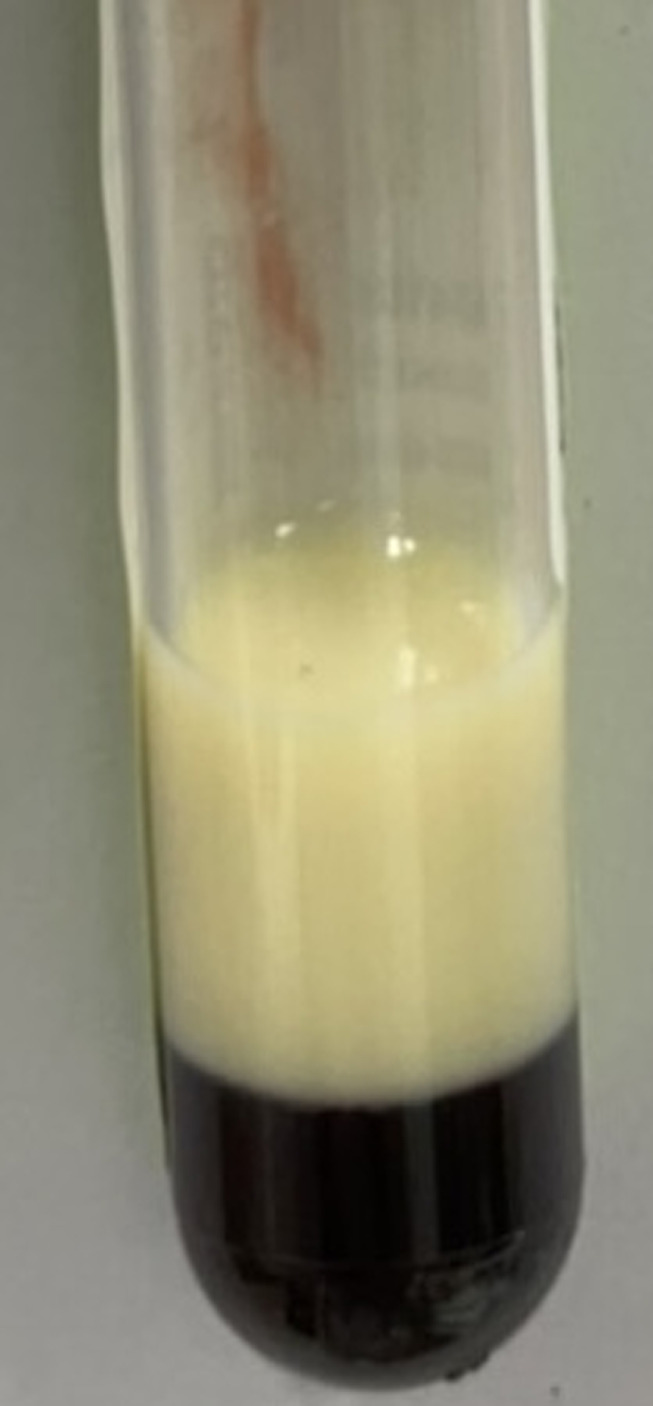
milky appearance of the blood sample

